# 3D Pose Detection of Closely Interactive Humans Using Multi-View Cameras

**DOI:** 10.3390/s19122831

**Published:** 2019-06-25

**Authors:** Xiu Li, Zhen Fan, Yebin Liu, Yipeng Li, Qionghai Dai

**Affiliations:** 1 Graduate school at Shenzhen, Tsinghua University, Shenzhen 518055, China; li.xiu@sz.tsinghua.edu.cn (X.L.); fanz14@mails.tsinghua.edu.cn (Z.F.); 2 Department of Automation, Tsinghua University, Beijing 100091, China; liuyebin@mail.tsinghua.edu.cn (Y.L.); daiqionghai@tsinghua.edu.cn (Q.D.)

**Keywords:** human 3D pose, closely interactive, two-stage assembling, multi-view images

## Abstract

We propose a method to automatically detect 3D poses of closely interactive humans from sparse multi-view images at one time instance. It is a challenging problem due to the strong partial occlusion and truncation between humans and no tracking process to provide priori poses information. To solve this problem, we first obtain 2D joints in every image using OpenPose and human semantic segmentation results from Mask R-CNN. With the 3D joints triangulated from multi-view 2D joints, a two-stage assembling method is proposed to select the correct 3D pose from thousands of pose seeds combined by joint semantic meanings. We further present a novel approach to minimize the interpenetration between human shapes with close interactions. Finally, we test our method on multi-view human-human interaction (*MHHI*) datasets. Experimental results demonstrate that our method achieves high visualized correct rate and outperforms the existing method in accuracy and real-time capability.

## 1. Introduction

Human pose detection is an active topic in the field of computer vision and computer graphics communities for many decades. Recovering human pose has a wild range of potential applications like human recognition and tracking, computer animation, mixed reality, automatic drive, etc. In the past few years, this problem has achieved remarkable progress due to the availability of CNN-based learning method for joints detection and connection. Based on the difference in human number and pose dimensionality, this problem could be divided into single person 2D pose estimation [1,2], single person 3D pose recovery [3,4], multi-person 2D pose detection [5,6] and multi-person 3D pose regression [7,8,9]. In this paper, we address the problem of multi-person 3D pose detection, specifically focusing on 3D pose recovering of closely interactive humans, which is common in real life such as dancing, hugging and boxing.

The problem of closely interactive humans 3D pose detection has not yet been extensively addressed due to large partial occlusion and truncation. Liu et al. [7] proposed a tracking method to solve this problem with manually fitting human skeleton and building human mesh by laser scan at first. This method heavily relies on the segmentation result which is a time-consuming process. With a strong assumption that the 2D poses of closely interactive humans have been detected correctly in every view image, Li et al. [10] introduced a spatio-temporal tracking algorithm to exploit spatial correspondence between views and temporal correlation among frames using multi-view videos as input. The main drawback of tracking method is error accumulation and drift since current pose estimation accuracy is heavily depending on previous results. Moreover, the tracking methods are usually initialized by ‘*human separate scenario*’ and get multi-person 3D poses and shapes based on single person pose recovery method. Different from tracking method, Joo H et al. [9] described a voting method for social interactive humans pose recovery based on a massive view system. This system includes more than 500 cameras which means it is not suitable for general pose detection task in natural scenes. Moreover, with sparse images as input, the voting method always fails to generate satisfying results due to the inherent ambiguities of 2D pose detection of closely interactive humans.

**Figure 1:**
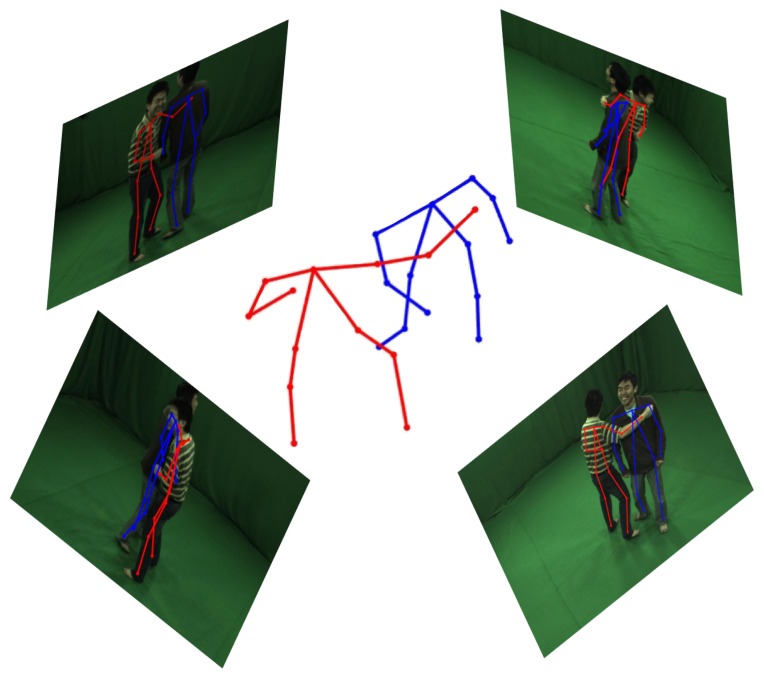
3D pose detection result of multiple markerless persons with close interactions from multi-view images at one time instance. Visualized results are shown as 2D poses in every view image reprojected from 3D pose.

In this paper, we proposed a two-stages assembling method to solve the problem of 3D pose estimation of closely interactive humans from sparse multiple view images at one time instance. Firstly, we adopt Openpose [5] to estimate 2D joints of each person in every image and find all valid joints which have high confidence score. Then, 3D joints are obtained by triangulation of corresponding 2D keypoints using an epipolar geometry proposed in [11]. After that, all possible 3D pose seeds could be assembled based on semantic meaning of every joint. In order to reduce human shape regressing time, a pre-assembling process is proposed to decrease pose seeds number. At last, we get the final result with post-assembling process. We test our algorithm on HMMI datasets generated in [7] and make a comparison with the state-of-the-art method proposed in [9]. Experimental results demonstrate that our method achieves a remarkable accuracy improvement compared with voting method [9]. Besides, the computation time of our method is much less than the state-of-the-art method (see Figure 1).

The main contributions of our work are summarized as the following three aspects: Firstly, a fully automatic method is proposed to detect 3D poses of closely interactive humans with sparse multi-view images as input. This method could reduce error accumulation efficiently without tracking process. Secondly, a novel interpenetration error function is introduced to minimize the intersections between human models. Lastly, a two-stage assembling architecture is present to improve the efficiency of our method.

## 2. Related Work

In recent years, human pose estimation has made great progress and achieved good performance with the growing popularity of CNN-based learning methods. Based on the different dimension of human pose result, existing works could be categorized into 2D pose detection and 3D pose and shape recovery.

### 2.1. Human 2D Pose Detection

As a basic element task of human pose estimation, joint detection has been broadly studied using data-driven learning method from one single unconstrained image. In 2014, multi-channel heat-maps had been proposed in [1] for the first time by Tompson et al., to represent key joint locations with per-pixel likelihood. Based on this, Stacked Hourglass [12] built a novel convolutional network architecture to improve the joint detection accuracy and CPM [13] achieved remarkable results with sequential architecture as well.

Multi-person 2D pose detection methods, as mentioned in Li et al. [10], could be divided into top-bottom approaches and bottom-top methods. Top-bottom approaches tend to convert multi-person pose estimation problem to single person pose detection task by cropping each person in the image [14]. For example, RMPE [6] proposed regional multi-person pose estimation framework to promote the human pose detection performance initialized by an inaccurate human bounding box. However, for humans with close interactions, proper bounding box for every person could be very difficult to get because of large part truncation between humans. In contrast, bottom-top approaches attempt to build connections among all detected joints within one image by defining different loss functions and various net architectures. OpenPose [5] obtained joint locations using non-maximum suppression and then, connected two associated joints belonged to one person by adopting part affinity fields (PAFs). DeepCut and DeeperCut [15,16] formulated the the partitioning and labeling problem with integer linear programming (ILP). Subsequently, Iqbal et al. [17] improved DeeperCut by adding the joint type obtained from CPM network to ILP. Moreover, associative embedding [18] proposed a innovational supervising convolutional neural network to group joints to individuals by encoding different humans with different tags.

### 2.2. 3D Pose and Shape Recovery

Three-dimensional pose and shape recovery could provide more information about human behaviors and the interactions between humans and surrounding environment. For single person 3D pose estimation, most existing methods were seeking for appropriate net architectures to lift 2D pose to 3D [3,19,20,21,22]. Besides, Rhodin et al. [23] proposed a multi-view image CNN learning method to estimate 3D human pose and annotate data automatically. SMPLify [24] is presented in 2016 by Bogo et al. to automatically estimate single person shape based on SMPL model [25] from one RGB image as input. To minimize the shape interpenetration error, SMPLify utilized capsules model learned from SMPL model. Kanazawa et al. [26] recover human mesh and pose through training an end-to-end net and outputting SMPL model parameters.

Multi-person 3D poses estimation approaches could be classified into single image learning method, multi-view videos tracking method and geometrical method taking multi-view images as input. Most generative *‘single image input’* approaches utilized CNN-based learning method to train an end-to-end model and obtained 3D pose result automatically. Similar to top-bottom approach in 2D pose detection from one single image, Rogez et al. [27,28] regressed multi-person 3D poses by LCR-net. After finding the bounding box for every person in one image, LCR-net turns this problem into single person 3D pose estimation task. Considering partial occlusions among persons, DensePose [8] took a full-blown supervised learning approach to get a surface-based human shape from an unconstrained image. Occlusion-Robust Pose-Maps (ORPMs) [29] were proposed to solve the invisible joint detection problem and output poses by connecting joints using PAFs as proposed in [5].

There has been substantial works taking multi-view videos as input and deriving multi-person 3D poses with tracking method. Here, we only investigate the works on the scenarios with closely interactive characters. Liu et al. [7] proposed a tracking method to capture the motion of markerless interactive humans with multi-view videos and human shapes as input. This work employed a maximum a-posteriori Markov random field (MAP-MRF) optimization framework for human instance segmentation in each image. It is a time-consuming process and heavily relied on the previous shapes estimated result. Meanwhile, multi-view human-human interaction (MHHI) datasets have been established in [7] which includes seven challenging motion sequences. Li et al. [10] designed a spatio-temporal tracker to find the continuous poses of humans with close interactions. They also estimated human shapes based on SMPL model proposed in [25]. This approach is strongly dependent on correct 2D pose detection result of every person, which is very difficult to get with tremendous occlusion and truncation between humans according to our experiments. Moreover, synthesized closely interactive 3D human pose-pairs datasets are generated by [30] with a Markov Chain Monte Carlo sampling method from a set of annotated 2D video frames.

Three-dimensional pose detection using multi-view images at one time instance could efficiently avoid error accumulation or drift compared with tracking method. Belagiannis et al. [31] addressed the problem of multi-person 3D pose estimation. They build 3D body part state space by triangulation corresponding 2D body part hypotheses. To resolve the problem of mixed body parts of multiple humans, the authors proposed 3D pictorial structure to represent human shapes. However, this method only considers the geometric cues to find 2D pose correspondences across every view. In crowded scenarios, it is not robust due to heavy occlusion and truncation. Joo et al. [9] built a massively multi-view system (Panoptic Studio) including more than 500 cameras to capture multiple human motions with social interactions. In this work, they first obtained score map for each joint using voxel grid voting method and got all 2D poses by CPM [13]. Then, a 3D voting method was introduced for part proposals to find the connections between joints based on the truth that the correct connection for one human part would result in the most views of the massive images. However, in the natural scene, it is impossible to build such an enormous system for image collection. Moreover, the voting method could not show a good performance in closely interactive person pose estimation task with sparse view cameras. To deal with 2D pose corresponding problem in multiple views, Dong [32] proposed a multi-way matching algorithm to find globally consistent correspondences. Besides, this method combined geometric and appearance information to calculate the affinity scores between bounding box in two views. However, this work also relies on single person bounding box and 2D pose detection results which could not guarantee the accuracy in close interactive scenarios.

## 3. Method

In this section, we first provide an overview of our method (Section 3.1), then present 3D joint triangulation loss function (Section 3.2). We describe in detail how we implement the pre-assembling process to reduce pose seeds (Section 3.3). Finally, we introduce our post-assembling optimization step to find the final result (Section 3.4).

### 3.1. Overview

**Figure 2:**
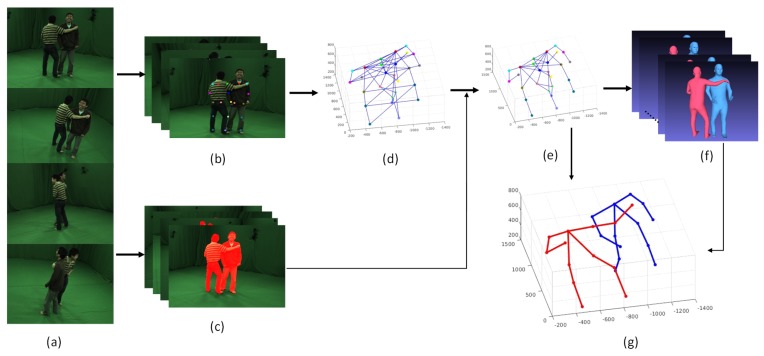
Method Overview. With multi-view images as input (**a**), we first detect the 2D joints (**b**) and human semantic segmentation (**c**) results with learning method in every view image. After triangulating 2D joints to 3D, we could get all 3D pose seeds (**d**) with all connection of semantic neighbor joints. Then we reduce 3D pose seeds number through pre-assembling process (**e**). At last, through pose-assembling optimization, the final 3D poses (**g**) could be obtained combined with SMPL models (**f**) fitting from pre-assembling pose seeds.

As shown in Figure 2, firstly, OpenPose is employed to get all visible 2D keypoints in every input image and human semantic segmentation results are acquired using MASK R-CNN; then, 3D joints are obtained by triangulation of the corresponding 2D keypoints with multi-view camera parameters; with all semantic 3D joints resulted from the above step, we transfer the pose detection problem to joints assembling task by optimizing the energy function shown in Equation (1). To reduce the pose seeds number for shape fitting process, we first group symmetry joints to one human based on the gray histogram of every joint and remove all illegitimate pose through pre-assembling process. After regressing all legitimate pose seeds to SMPL model, we optimize the post-assembling functions with all shapes using the last three terms and output the final pose result.

We denote the bold notation $J=\{J_{t}^{h}\}$ as assigned 3D human joints where $J_{t}^{h}\in R^{3}$ is the *t*-th type joint of the *h*-th person. With the same index h, $J^{h}$ represent the 3D pose for person h. Here, we employ COCO_19 joint definition and only use 13 body joints (including neck, shoulders, elbows, wrists, hips, knees and ankles) for one person and set T as 13. $J=\{J_{t}^{m}\}$ is unallocated 3D joints set and $J_{t}^{m}$ is the *m*-th point of type *t* joint. 2D joints are notated as $k=\{k_{tc}^{m}\}$ where $k_{tc}^{m}\in R^{2}$ means one 2D joint in view c. Apart from joint definition, we indicate human part with symbol $P=\{P_{n}^{h}\}$ where *n* = 1,2, … N and N is the number of part of one person (N = 12 in COCO_19) and $p=\{p_{nc}^{h}\}$ is the 2D skeleton in every view image. Since our method also leverages human shape information, we adopt $S=\{S_{h}\}$ to represent the fitting shapes of all persons. *P* and *S* are calculated from **J** and *p* is reprojected from *P*.

(1) $$\begin{array}{c} L\left(J\right)=\;\underset{J}{\arg\min}\left\{\;\;\overset{post-assembling}{\overset{︷}{\omega_{D}D\left(J,J_{S}\right)\;+\;\omega_{O}O\left(S\right)\;+\;\omega_{R}R\left(P\right)}}\;\right\}\\ s.t.\;\;\underset{pre-assembling}{\underset{︸}{U(p)\leq 1,\;H(J)\leq 1,\;L(P)\geq 1\;}}. \end{array}$$

*U* is imported to make sure that the human skeleton is always in ’human’ label in semantic segmentation image. Furthermore, *H* is used to constrain that one kind joint could only be assigned on one person. *L* is the term that encourages the symmetry part skeleton length to be equal. The above three terms compose pre-assembling process leveraged to reduce the searching range of pose-assembling step based on human mesh. Using the fact that the right assembling result should fit the regressed model well, *D* is the first post-optimizing term used to minimize distance error of the input 3D joints and output joints on every human shape. With the human model fitting results, interpenetration constrain *O* could make sure that human shapes contain the minimum overlaps. Similar to the joint color grouping process, *R* is imported to guarantee the similarity of symmetry part gray histogram in 3D shapes.

### 3.2. 3D Joints Triangulation

In recent years, 2D joints detection in one image has achieved remarkable results [5,12,13]. In this paper, we use OpenPose [5] to get joint detection results represented as confidence maps. After performing non-maximum suppression, every joint is described as a certain location with a confidence score s. However, we found that some invisible keypoints could also be detected, so a score threshold $\tau_{s}$ is set to remove the keypoints which contain lower confidence score than $\tau_{s}$.

We triangulate the 3D position of every joint using enumerating epipolar geometry [11] after finding all the corresponding keypoints with the same semantic meaning in every view image. The minimum reprojection loss function used for triangulation is defined as

(2) $$\Phi \left(J_{t}\right)=\underset{J_{t}}{\arg\min}\sum_{c=1}^{C}\sum_{m=1}^{M}v_{k}\left(k_{tc}^{m}\right){∥P_{c}\left(J_{t}^{m}\right)-k_{tc}^{m}∥}_{2}$$

Here, $J_{t}\in R^{3\times M}$ means all 3D coordinates of the *t*-th type kind joint and $P_{c}\left(J_{t}^{m}\right)$ is the reprojections of joint $J_{t}^{m}$ in view c. $v_{k}\left(k_{tc}^{m}\right)\in \left\{0,1\right\}$ means the visibility of $k_{tc}^{m}$. To avoid expensive computation in all human joints enumeration step, for one kind joint, we take two view images first which contain the most *t*-th joints and triangulate the 2D joints to 3D. Then, we find the corresponding points on rest views and calculate the rest joints with the same method for other un-triangulated keypoints. Taking two closely interactive persons as an example, we pick two view images which take four *t*-th joints totally and output two 3D joints through minimizing reprojection error. If there are only three *t*-th joints on two views, we could triangulate one joint and find the corresponding keypoints in other views and then calculate another 3D joint with the rest of the un-triangulated keypoints in every view which are corresponded obviously.

### 3.3. Joints Pre-Assembling

It is a time consuming process if we regress all pose seeds generated from enumerate method into human shape. So, to improve the method efficiency, we first employ symmetry joints grouping and pre-assembling step to reduce the pose seeds number.

#### Symmetric Joints Grouping

**Figure 3:**
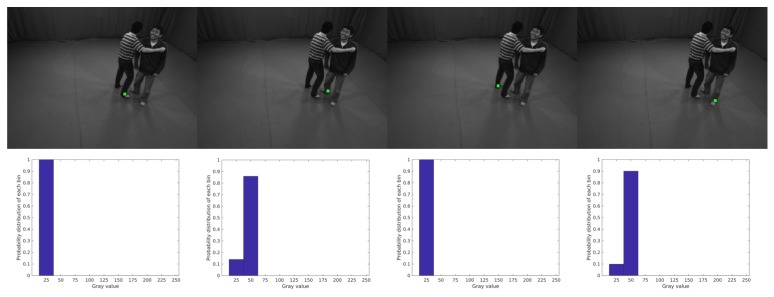
Symmetry Ankle Joints Grouping.

Based on the fact that the gray distribution of symmetric joints on one human should be similar, we could group corresponding joints to one person through the comparison of symmetric joint gray histograms. As shown in Figure 3, taking ankle joints for an example, there are four ankle joints for two persons and two symmetry joint combinatorial results: $G_{1}=\left\{G_{11},G_{12}\right\}=\left\{\left\{LA1,RA1\right\},\left\{LA2,RA2\right\}\right\}$ and $G_{2}=\left\{G_{21},G_{22}\right\}=\left\{\left\{LA1,RA2\right\},\left\{LA2,RA1\right\}\right\}$. For one kind of joint, we first build a gray distribution histogram based on the patch around every joint. Then, the BinNum is set to 10 to decrease the illumination effect and only concern the probability of every BinValue. Here we leverage Bhattacharyya coefficient Bh on every pair of symmetry joints to measure the similarity of their gray probability distributions. In our example, the Bhattacharyya coefficient result is $BH_{1}=\left\{Bh\left(LA1,RA1\right),Bh\left(LA2,RA2\right)\right\}=\left\{0.9979,0.9657\right\}$ and $BH_{2}=\left\{Bh\left(LA1,RA2\right),Bh\left(LA1,RA2\right)\right\}=\left\{0.3012,0\right\}$. Then, the ratio of mean Bhattacharyya coefficient of two combinatorial is $\left(min\left(mean\left(BH_{1}\right),mean\left(BH_{2}\right)\right)/max\left(mean\left(BH_{1}\right),mean\left(BH_{2}\right)\right)\right)<\tau_{G}$, where $\tau_{G}=0.5$ is the threshold value indicating the distinct gray distribution difference and we could get the grouping result $G_{1}$ for ankle joint.

#### Skeleton Reprojection Term *U*

It is rational to assume that the correct assembled 2D skeleton p should always be in ’human’ region. Here, we define a skeleton reprojection term to force the 3D skeleton projections in every view image labeled as ’human’ in semantic segmentation images. Learning methods [14,33] have provided fantastic segmentation results especially in ’human’ label benefit from massive human datasets. In this paper, we utilize MASK R-CNN to provide accurate segmentation results and the skeleton reprojection term *U* is defined as:

(3) $$U\left(p\right)=\sum_{n=1}^{N}\sum_{c=1}^{C}\sum_{h=1}^{H}v_{p}\left(p_{nc}^{h}\right)\cdot u\left(p_{nc}^{h},SI_{c}\right)$$

where $v_{p}\left(p_{nc}^{h}\right)=v\left(k_{t_{a}c}^{h}\right)\cdot v\left(k_{t_{b}c}^{h}\right)$ is the part visibility with the definition that part $p_{nc}^{h}$ is connected by keypoints $k_{t_{a}c}^{h}$ and $k_{t_{b}c}^{h}$. $u(p_{nc}^{h},SI_{c})$ is the ’non-human’ point number of skeleton line segment $p_{nc}^{h}$ in semantic segmentation image $SI_{c}$.

#### Symmetry Limb Length Constrain *L*

With the semantic meaningful joints assigning to one human, the symmetry limbs of a legitimate human pose should have equal lengths. Considering the inaccuracy of 2D joints detection from CNN-net, we use the symmetry part length term *L* to get rid of significantly failed assembling results. The symmetry part length constrain *L* is defined as:

(4) $$\begin{array}{c} L\left(P\right)=\prod_{n=1}^{6}\prod_{h=1}^{H}r_{n}^{h}\\ where\;\;r_{n}^{h}=\left\{\begin{array}{c} 1\;,\;\;\;\;\frac{min(LP_{n1}^{h},LP_{n2}^{h})}{max(LP_{n1}^{h},LP_{n2}^{h})}-\tau_{L}>0\\ 0\;,\;\;\;\;others \end{array}\right. \end{array}$$

Here, $\tau_{L}$ is a threshold value we set to remove the pose with illegal symmetry part length and $LP_{n1}^{h}$ and $LP_{n2}^{h}$ are the length of a pair of symmetry parts $P_{n2}^{h}$ and $P_{n1}^{h}$. $r_{n}^{h}$ will be set to 1 if $LP_{n1}^{h}\cdot LP_{n2}^{h}=0$.

### 3.4. Joints Post-Assembling

After the pre-assembling process, we could get a pool of combination poses which contains the truth assembling result. For every pose seed, we regress it to human shape using skinned multi-person linear (SMPL) model [25]. SMPL model outputs a triangulated surface with 6980 vertices calculated from a function M(β;θ;γ). β,θ and γ are shape parameters, pose parameters and model parameters respectively which are represented as vectors. Besides, model joint locations could be calculated from parameter β with the function $J_{s}=B\left(\beta \right)$. It is reasonable to believe that the correct pose input should take a small distance error with the model output pose. We use L2-norm to define the fitting joint distance error:

(5) $$D\left(J,J_{s}\right)=\sum_{t=1}^{T}\sum_{h=1}^{H}v_{J}\left(J_{t}^{h}\right){\left\|J_{t}^{h}-{\left\{J_{s}\right\}}_{t}^{h}\right\|}^{2}$$

#### Shape Interpenetration Constrain *O*

In closely interactive scenarios, human shapes fitting from the wrong poses will always contain intersections. As mentioned in [24], existing methods always approximate body surface using proxy geometries [31,34,35]. Bogo et al. adopted learning method to find a regressor to approximate bodies with capsules from SMPL model. This method could improve the interpenetration result efficiency. However, compared with the original model, this is also a proximate result based on lots of training data which is hard to get. In this paper, we propose an efficient and more accurate method to get the model intersection degree based on the human mesh resulted from the SMPL fitting process.

For two intersecting human parts from different persons, the two line segments of part skeletons should have common perpendicular and the largest intersection should be located in the orientation of common perpendicular. Here, we calculate the overlap score of two parts through the comparison between common perpendicular length and part radiuses on the foots of perpendicular. To easily describe the torso skeleton, we import a virtual joint call “*root joint*” which is located at the center of left and right hip joints and the connection between root joint and neck joint is defined as the skeleton line segment of torso part. Besides, we label every mesh point of SMPL model from 1 to 9 to indicate every different part of one human. The definition of shape interpenetration constrain is shown in Equation (6)

(6) $$O\left(S\right)=\max{\left\{IPP_{i,j}\right\}}_{j=1,2\ldots 9}^{i=1,2\ldots 9}$$

$IPP_{i,j}$ is the interpenetration degree of part *i* and part *j* from two humans, which is defined as:

(7) $$IPP_{i,j}=\exp\left(-\frac{L_{cp}^{ij}}{MR_{i}+MR_{j}}\right)$$

Here, $L_{cp}$ is the length of common perpendicular between skeleton line segments of part *i* and part *j*. $MR_{i}$ and $MR_{j}$ are the mesh radius in the direction of common perpendicular.

#### Symmetry Parts Gray Similarity Term R

Since we leverage the general SMPL model to approximate human body surfaces, it is inevitable that even the shapes fitted from correct poses will also contain some overlaps. So, with the shape interpenetration constrain *O*, the incorrect results which have no shape collisions will be output sometimes. To solve this problem, we introduce the symmetry parts gray term R to encourage the symmetry part mesh of one person should always take similar gray distributions. Due to the occlusion between the closely interactive humans, it will be hard to get the color distribution in one image even with the human shape reprojected to original images. So, we employ Hidden Point Removal (HPR) algorithm [36] to get the visibility of every mesh point in every view without reconstructing a surface or estimating normals. Through our test, the best performance can be obtained when the radius parameter of HPR is set as 3. Then, the probability distribution of gray value for every part is estimated by calculating of image intensity histogram reprojected from visible mesh points. Lastly, we measure the gray similarity of the symmetry part for each person using Bhattacharyya distance on gray probability distributions similar to symmetry joints grouping process.

## 4. Results

In this part, we first introduce multi-view human-human interaction (MHHI) datasets [7] and test our method on four kinds of interaction motions (Section 4.1). Then, the comparison results with state-of-the-art multi-person 3D pose detection method are shown in Section 4.2. At last, the experiment environment and method efficiency is discussed in Section 4.3.

### 4.1. Datasets and 3D Pose Detection Results

Multi-view human-human interaction (MHHI) datasets [7] are recorded by 12 synchronized and calibrated cameras with the image resolution of 1296×972. There are seven challenging motion sequences (Crash, Dance, Double, Fall, Fight, Jump and ShakeHug) in the datasets and each motion sequence contains about 230 frames with two humans in the scenes. In this paper, we test our method using four published motion sequences including Crash, Double, Fall and Fight. It is worth mentioning that HMMI datasets include pre-closely-interactive and after-closely-interactive scenes which could be easily separated into single person problem. Our experiments are only focused on motions with closely interactive features which contain about 235 poses in four sequences totally. For every instance, we take four view images as input and evaluate the results with visualized 2D pose by reprojecting the estimated 3D pose to original images.

**Figure 4:**
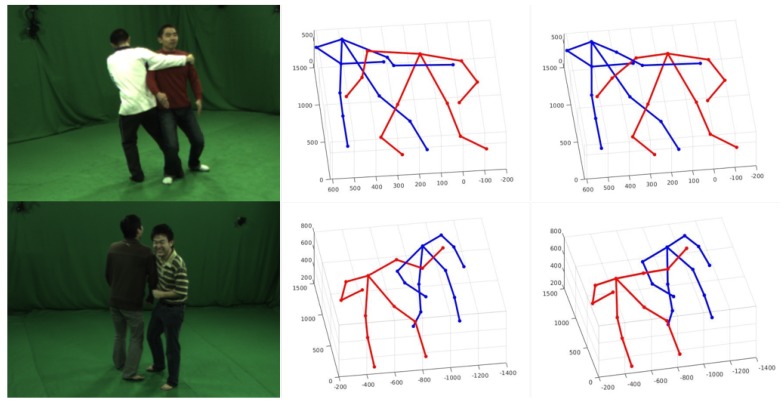
Comparison results for 3D pose detection without (**Middle**) and with (**Right**) interpenetration constrain O.

**Figure 5:**
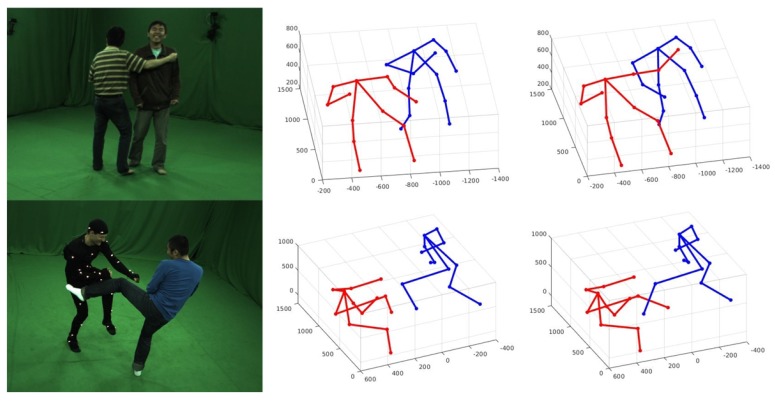
Comparison results for 3D pose detection without (**Middle**) and with (**Right**) symmetry parts gray term R.

**Figure 6:**
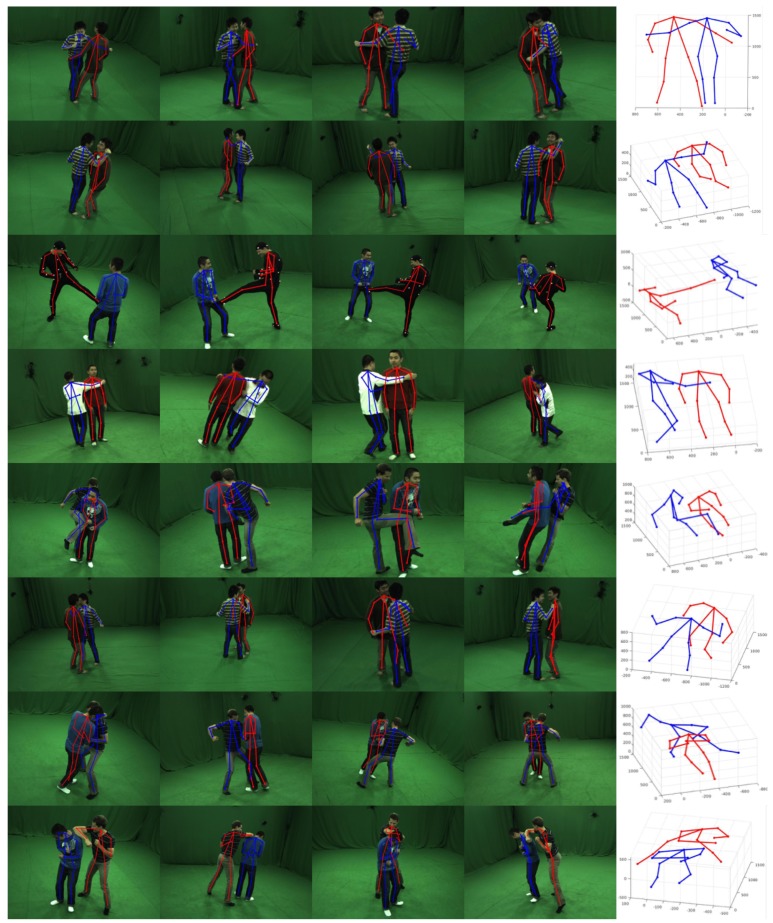
3D pose detection results on MHHI datasets. The first four columns are the 2D pose results reprojected from 3D pose result. The last column is the 3D pose estimation result of our method.

To investigate how the main components in our pipeline affect the detection results, we perform an ablation study for interpenetration constrain O and symmetry parts gray term R. We firstly explore the impact of interpenetration constrain O and the result is shown is Figure 4. Intersection problems of human shapes fitting from the wrong poses will occur frequently in closely interactive scenarios. When the same meaning joints from different humans are very close, the assembling process without interpenetration constrain O will be confused to get the right assignment of those keypoints. Through our experiment, the correct rate will drop to 72.6% due to the absence of interaction avoiding term O. Figure 5 shows some incorrect 3D poses detection results without symmetry parts gray constrain R. The mean correct rate of pipeline without this term is about 81.9%. The false results always contain the minimum interpenetration between human models and those mistakes could be avoided when we force the gray distributions of symmetry parts on one human to be similar. The results from our complete method are shown in Figure 6. The first four columns are 2D reprojected pose results in different views and the last column is 3D pose result using our method. It can be seen that our method could output the correct pose of markerless humans with close interactions and the correct accurate is about 93.2%.

### 4.2. Comparison

**Figure 7:**
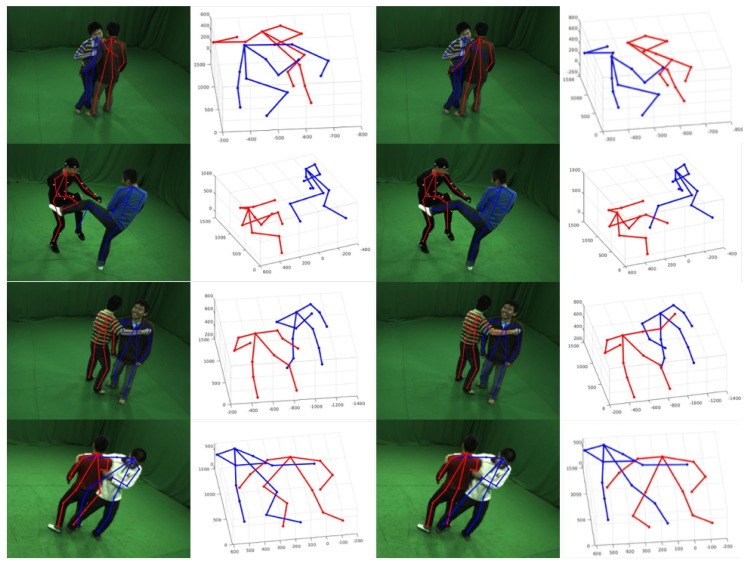
Comparison results with voting method. The first two columns are the pose detection results using voting method. Results of our method are shown in the last two columns.

To our best knowledge, there is no work to deal with the problem of 3D pose detection of closely interactive humans with sparse multi-view images without tracking. The most relevant work is proposed by Joo [9] which built a massive multi-view system to capture 3D poses of social interactive humans. With the assumption that the correct 2D pose should occur at most view images, Panopic Studio proposed a voting method to get part proposals and generated skeletal proposals by dynamic programming. Figure 7 shows the comparison results using four different view images as input. The first two columns are the 3D pose detection results from Panopic Studio and one 2D pose reprojected on the original image, and the last two columns are our results. Through our test, CNN-based methods for 2D pose detection make it easy to establish the wrong connection between two semantic adjacent joints at the closely interactive parts. Due to the sparse views we use, the voting method will fail and output the wrong 3D pose result. It can be seen that our method is more robust when handling occlusion and complex people interactions than [9].

#### View Selection Analysis

2D joints detection and pose estimation results from learning method are easily influenced by view selection arguments. To investigate the affection of view selection strategy to our method and the baseline solution proposed in [9], we design two experiments to analyze the impact of multiple view positions and camera numbers separately.

**Figure 8:**
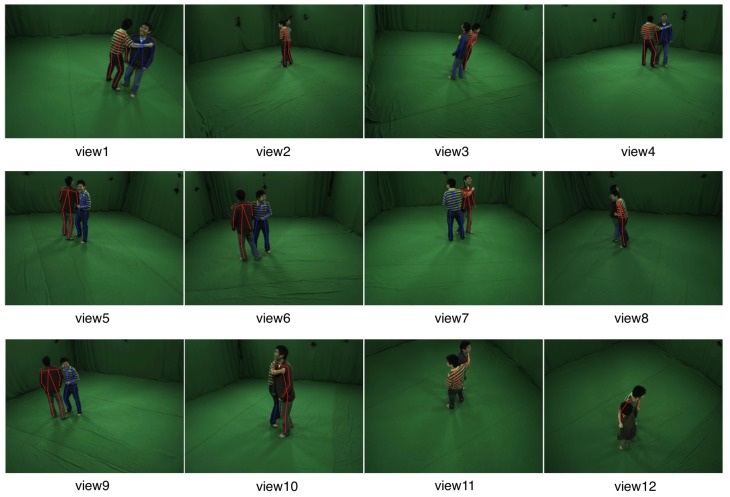
Positions of 12 Camera Views.

**Figure 9:**
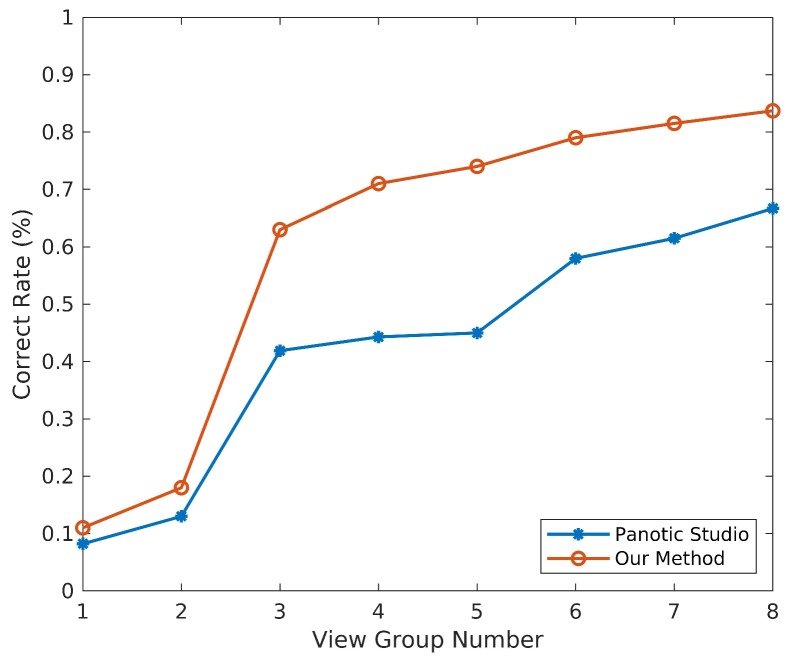
Results of different view groups.

For view position evaluation, we take the fixed camera number as 3 and the position of 12 available cameras is shown in Figure 8. Eight kinds of deterministic selection of camera groups from 12 views have been set in Table 1. The correct rate distribution of different view selection group is given in Figure 9. It can be seen that the correct rates of our method and the voting method [9] both are improved with the increasing distribution uniformity of selected cameras. With large occlusion and truncation in close interactive scenes, the proper relative position of selected views could provide more 2D cues such as joints or poses, which are needed for 3D motion capture. Besides, we also make a test for random selections of three views and the mean correct rate of our method is about 62.3% and 37.4% for [9].

**Figure 10:**
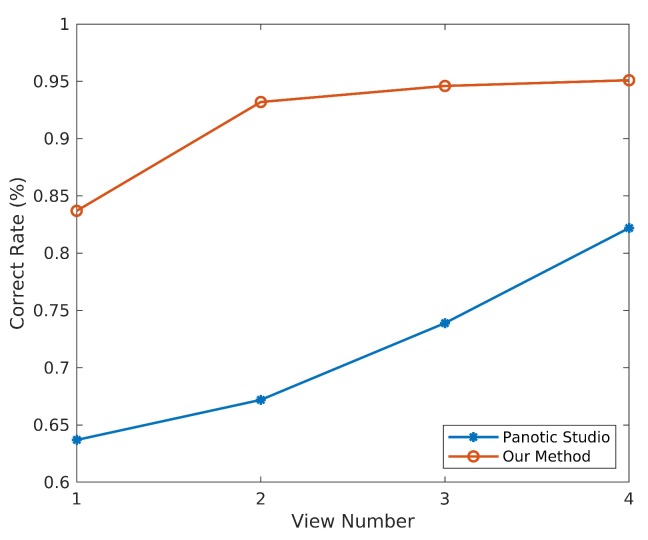
Results of different view numbers.

Then, we select cameras with proper relative position and explore how the 3D pose detection result is effected by the number of camera views. As shown in Figure 10, it can be observed that the detection result becomes better as the number of camera views increases. From our test, our method could output the right 3D pose for closely interactive humans with at least three views which have appropriate relative poses. With the same arguments of camera views selection, the assembling method proposed by us could outperform the voting solution.

### 4.3. Method Efficiency

We use a machine with 4-core Intel i7-6700k 4 GHz CPU, 32 G RAM and one GPU of NVIDIA TITAN X. GPU is only used at 2D joint detections and human semantic segmentation processes. These two parts cost about 0.2 s per frame totally. The first optimization state takes about 5.3 s and the model loading process of SMPL fitting takes about 2.6 s. Depending on the number of 3D pose seeds after first optimization state, the mean time consumption of the second optimization stage is about 12.4 s. We optimize our algorithm with adding a maximum pose seeds number constraint for the pre-assembling state results and the computation time of the pose-assembling stage could decrease to 4.1 s on average. The total running time of our method for one time instance is about 14 s. We also test the time consumption with non-two-stage method which means fitting SMPL model to about 4096 pose seeds and it will takes more than five minutes per frame on average.

## 5. Conclusions and Future Work

In this paper, we propose a 3D pose detection method of makerless humans with close interactions from sparse multi-view images at one instance. To improve the efficiency of our method, we present a two-stage assembling method to reduce the 3D pose seeds before fitting to the SMPL model which is used in the pose-stage assembling process. We also introduce a new method to evaluate the shape interpenetration degree which is an important constrain of our method. We test our method on challenging HMMI datasets. An ablation study is performed to prove the importance of the main component in our pipeline. Experimental results demonstrate that our method performs better than the state-of-the-art approach.

There are three points in future work to expand our work. Firstly, we will try to solve the shooting angle problem using multiple intelligent flying cameras to capture images at appropriate views which is studied on single person by [37,38] preliminarily. Secondly, we will refine our 3D pose result with a training valid pose model constrain to minimize the pose error due to the inaccuracy of 2D joint detection results. Lastly, we will improve the efficiency of our method by using GPU acceleration.

## Figures and Tables

**Table 1:** Deterministic selection of different view groups.

Group Number	Camera Number	Group Number	Camera Number
1	cam8, cam11, cam12	2	cam3, cam10, cam12
3	cam1, cam4, cam7	4	cam5, cam6, cam9
5	cam1, cam2, cam3	6	cam4, cam9, cam10
7	cam1, cam6, cam8	8	cam5, cam7, cam10
